# A new *Chorizococcus* species (Coccoidea, Pseudococcidae) from Taiwan with transferring of *Chorizococcus mirzayansi* Moghaddam to genus *Spilococcus* Ferris

**DOI:** 10.3897/zookeys.103.1215

**Published:** 2011-06-10

**Authors:** Ming-Yu Tsai, Wen-Jer Wu

**Affiliations:** 1 Department of Entomology, National Taiwan University, Taipei 106, Taiwan; 2 Research Center for Plant Medicine, National Taiwan University, Taipei 106, Taiwan

**Keywords:** *Chorizococcus zoysiae* sp. n., *Zoysia tenuifolia*, Pseudococcidae, Taiwan, *Spilococcus mirzayansi* comb. n.

## Abstract

A new mealybug species, *Chorizococcus zoysiae* **sp. n.**, feeding on *Zoysia tenuifolia* (Poaceae), is described from Taiwan. Adult female, third-instar female, second-instar female and first-instar nymph were described and illustrated in this article. Keys are provides to (a) separate this new species from similar species of *Chorizococcus* and those of same genus on zoysia grasses and (b) to identify instars of the new species. In addition, *Chorizococcus mirzayansi* Moghaddam is transferred to the genus *Spilococcus* as *Spilococcus mirzayansi* (Moghaddam), **comb. n.**

## Introduction

Zoysia grasses (*Zoysia* spp.) are creeping grasses native to southeastern and eastern Asia and Australasia. Because of high tolerance to temperature, sunlight, and water, they are widely used for lawns in different climate regions. According to Ben-Dov et al. (2010), there are nine scale insects found on zoysia grasses, and five of them belong to the family Pseudococcidae. Among these species, *Chorizococcus rostellum* (Lobdell) was found on an undetermined *Zoysia* species.

The mealybug genus *Chorizococcus* includes 56 described species, half of them from the Nearctic region and others from another five regions (Ben-Dov et al. 2010). Although the genus is of worldwide distribution, many of the species are restricted to a limited area, except *Chorizococcus rostellum* which is distributed in four biogeographic regions. Althoughsix *Chorizococcus* species are known from the Oriental region and occurred in India and Sri Lanka, so far there is no species have been reported from Southeast Asia. This genus has been reported on 117 plant species belonging to 25 plant families. Among them Poaceae and Asteraceae are major host plant families (Tang 1992, Ben-Dov 1994, Ben-Dov et al. 2010, Fallahzadeh et al. 2010, Moghaddam 2010). There are very specific discussions on the genera *Chorizococcus*, *Spilococcus* and *Vryburgia* on cerarii numbers and other characters of these genera. In the other hand, the generic status of *Chorizococcus mirzayansi* Moghaddam is questionable due to the number of cerarii, and some taxonomic works should be made.

In this paper, we provide taxonomic descriptions and illustrations of the adult female, third-instar female, second-instar female and first-instar nymph of a new *Chorizococcus* species. In addition, a key is provided to separate this new species from *Chorizococcus kandyensis* and *Chorizococcus rostellum* which are occurred around the Southeast Asiaand another key is proposed for distinguishing different stages of this new species. We here transfer *Chorizococcus mirzayansi* Moghaddam to the genus *Spilococcus* as *Spilococcus mirzayansi* (Moghaddam) concerning to the original description of this species.

## Materials and methods

All mealybug specimens were slide-mounted in Euparal using the method outlined in Williams and Granara de Willink (1992), except that xylene was used instead of clove oil.

The morphological terms used in the descriptions are explained by Williams (2004) and Williams and Granara de Willink (1992). The descriptions and measurements are based on more than 10 slide-mounted specimens, all in good condition, except for second instar female and first instar nymph of which eight and four specimens were available, respectively. All measurements are given as minimum and maximum. Holotype measurement is also provided in parentheses. Tarsal lengths excluded the claw. Setal lengths included the setal base. Each figure shows a generalized individual based on some specimens used for the description. The enlargements around the central drawing are not drawn to the same scale as each other.

All type specimens of the new species are deposited at Department of Entomology, National Taiwan University, Taipei, Taiwan (NTU), except for three female paratypes deposited in the insect collection of the Taiwan Agricultural Research Institute, Wufeng, Taichung, Taiwan (TARI), and another three female paratypes deposited at the Natural History Museum, London, UK (BMNH).

## Taxonomy

### Key to Chorizococcus species on zoysia grasses and Ch. kandyensis

**Table:** 

1	Circulus present	2
–	Circulus absent	*Chorizococcus zoysiae* sp. n.
2	Ventral oral rim tubular ducts present; many dorsal oral rim tubular ducts present and extend from marginal areas to medial areas of head and thorax	*Chorizococcus kandyensis*
–	Ventral oral rim tubular ducts absent; seldom dorsal oral rim tubular ducts present in marginal areas of head and thorax only	*Chorizococcus rostellum*

### Key to instars of Chorizococcus zoysiae sp. n.

**Table:** 

1	Antennae 6-segmented	2
–	Antennae 7- or 8-segmented	3
2	Oral rim tubular ducts present	second-instar female
–	Oral rim tubular ducts absent	first-instar nymph
3	Antennae 7-segmented; without multilocular disc pores; without vulva	third-instar female
–	Antennae 8-segmented; with multilocular disc pores; with vulva	adult female

#### Chorizococcus

McKenzie, 1960

http://species-id.net/wiki/Chorizococcus

Chorizococcus McKenzie 1960: 692; 1967: 86; Miller and Mckenzie 1971: 569; Williams 1970: 124; Williams, 1985: 75; Williams 2004: 106; Williams and Watson 1988: 31; Williams and Granara de Willink 1992: 100; Ben-Dov 1994: 82.

##### Diagnosis.

Body elongate oval to broadly oval; membranous. With 1–5 pairs of cerarii present on posterior segments of abdomen and sometimes a pair on head also, each cerarius bearing 2 conical setae; auxiliary setae present on anal lobe pair only. Oral rim ducts, sometimes of 2 sizes, present on dorsum and commonly also on venter. Oral collar tubular ducts present, at least on venter; if present on dorsum, then restricted to marginal areas. Antennae each with 7 or 8 segments. Legs well developed, with translucent pores on hind coxae, sometimes absent. Claw stout, without a denticle. Claw digitules knobbed. Tarsal digitules minutely knobbed. Multilocular disc pores present on venter, rarely found on dorsum. Circulus present or absent, when present usually divided by intersegmental line. Anal ring normal, bearing 6 setae. Anterior and posterior ostioles present.

##### Comments.

*Chorizococcus* McKenzie comes close to *Vryburgia* De Lotto in possessing dorsal oral collar tubular ducts. Oral collar tubular ducts on dorsum in *Chorizococcus* occur on margin only, whereas in *Vryburgia*, those ducts occur in transverse rows on dorsum (Williams 2004). *Spilococcus* Ferris is almost identical with *Chorizococcus* McKenzie but possesses 6–17 pairs of cerarii, six or more of which present on the abdomen. Miller and Mckenzie (1973) discussed the difficulties of assigning species to either *Chorizococcus* or *Spilococcus*. Danzig (1998) did not accept *Chorizococcus* and included all species with 1–17 pairs of cerarii in *Spilococcus* *sensu lato*. Here we follow the definition of Wiliiams (2004) and accept the usage of *Chorizococcus* McKenzie.

#### Spilococcus mirzayansi

(Moghaddam, 2010) comb. n.

http://species-id.net/wiki/Spilococcus_mirzayansi

Chorizococcus mirzayansi Moghaddam 2010: 64.

##### Comments.

 According to the descriptions of *Chorizococcus mirzayansi*, it should not be placed in genus *Chorizococcus* based on possessing more than 5 pairs of cerarii, a major diagnostic character in distinguishing genus *Chorizococcus* from genus *Spilococcus* in Williams’s definition (2004) or it should be included in genus *Spilococcus sesu lato* proposed by Danzig (1998). A new combination is made here for *Chorizococcus mirzayansi* as above.

#### Chorizococcus zoysiae

Tsai sp. n.

urn:lsid:zoobank.org:act:F243D130-D94E-4650-B741-5AD0FB364531

http://species-id.net/wiki/Chorizococcus_zoysiae

##### Material studied.

 **Holotype: adult female,** **Taiwan,** Tainan City, East district, on leaf sheaths of Korean velvet grass(*Zoysia tenuifolia*), 7.XI.2006, S. K. Chen (NTU): 1/1 in good condition.

##### Paratypes:

 13 adult females, 13 third-instar females, 8 second-instar females, 4 first-instar nymphs, same data as holotype (NTU); 3 adult females, same data as holotype (TARI); 3 adult females, same data as holotype (BMNH).

##### Description of the Adult Female.

**Figure 1.**
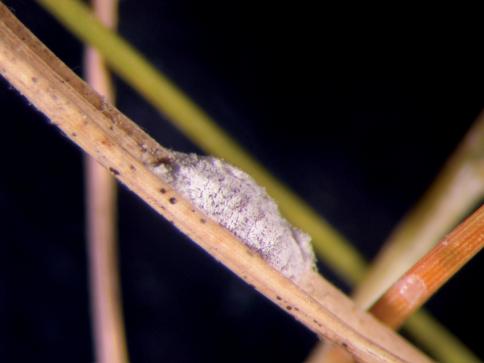
Live adult female of *Chorizococcus zoysiae* Tsai, sp. n., on *Zoysia tenuifolia*.

 **Field Features.** Body color dark-brownish, covered with thin white mealy wax. Adult female and older nymphs with white, filamentous wax secretion resembling “fur” and covering both mealybug and host plant (Fig. 1). All stages of this mealybug stayed beneath the leaf sheath.

**Figure 2.**
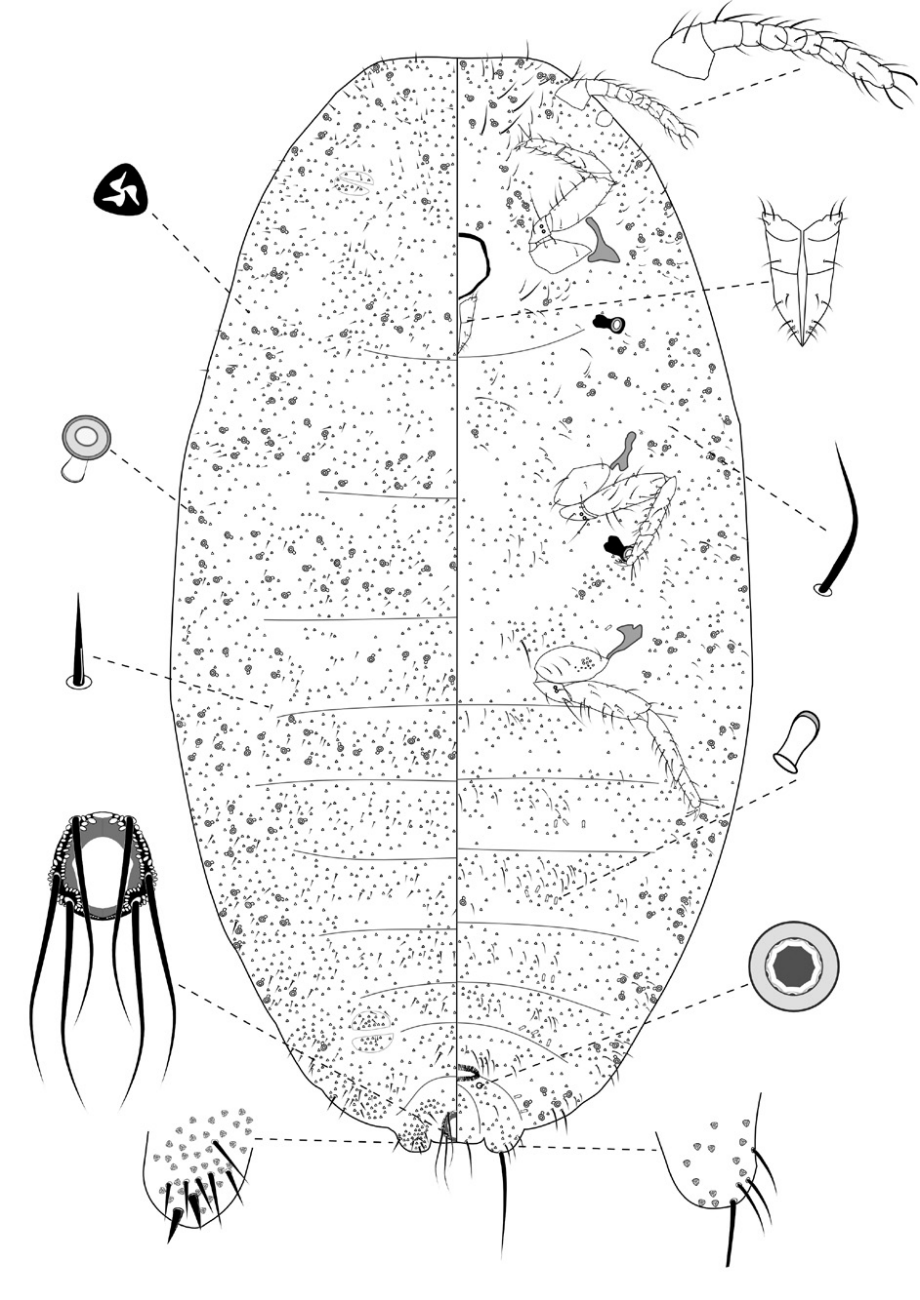
Adult female of *Chorizococcus zoysiae* Tsai, sp. n.

**Slide-Mounted Features** **(measurements based on 20 specimens).** Body elongate oval, 1.57–2.56 (2.44) mm long and 0.76–1.33 (1.17) mm wide (Fig. 2). Antennae each 233–267 (247) μm long, with 8 segments, occasionally 7 segments. Clypeolabral shield about 119–151 (144) μm long. Labium about 111–122 (117) µm long, shorter than clypeolabral shield. Legs well developed, stout; fore legs: coxa ca. 61–111 (94) µm long, trochanter + femur 206–233 (217) µm long, tibia + tarsus 178–206 (194) µm long, ratio of lengths of tibia + tarsus to trochanter + femur 0.81–0.95 (0.90), ratio of lengths of tibia to tarsus 1.49–2.00 (1.69); mid legs: coxa ca. 94–117 (97) µm long, trochanter + femur 222–250 (217) µm long, tibia + tarsus 189–217 (194) µm long, ratio of lengths of tibia + tarsus to trochanter + femur 0.81–0.90 (0.83), ratio of lengths of tibia to tarsus 1.64–2.09 (1.96); hind legs: coxa ca. 97–133 (111) µm long, trochanter + femur 239–269 (256) µm long, tibia + tarsus 217–250 (244) µm long, ratio of lengths of tibia + tarsus to trochanter + femur 0.87–0.98 (0.96), ratio of lengths of tibia to tarsus 1.78–2.50 (2.14); claw stout, without denticle, about 25–26 (25) µm long. Translucent pores numbering 11–20 (13), present on posterior surface of hind coxa. Circulus absent. Both anterior and posterior ostioles present, not well developed, each lip with a few trilocular pores and 1–4 setae. Cerarii numbering usually 2 pairs, situated on posterior abdominal segments, occasionally 3 pairs. Anal lobe cerarii (C18) each bearing 2 slender conical setae, each seta about 18–25 (21) µm long, with 6–9 auxiliary setae and 16–20 trilocular pores. Penultimate cerarii (C17), each containing 2 slender conical setae, about 15–22 (19) µm long, and few triocular pores. Antepenultimate cerarii (C16), if present, each bearing 2 slender conical setae, about 14–18 (16) µm long, and few trilocular pores. Each cerarius situated on a membranous area. Anal lobes moderately developed, each ventral surface membranous, bearing an apical seta 111–144 (139) μm long. Anal ring about 46–68 (62) µm long and 57–73 (65) µm wide, bearing 6 setae, each seta about 99–138 (112) µm long.

**Dorsum.** Dorsal surface with slender but stiff setae present, mostly each 13–28 (18) µm long, associated by shorter setae of different sizes, about 5–7 (7) µm long, except for longer setae on abdominal segment VI and VII, each about 35–38 (36) µm long. Triocular pores present, each ca. 4 µm in diameter, scattered over dorsum. Multilocular disc pores absent. Oral rim tubular ducts, each with rim about 6–10 (6) µm in diameter and tube about 6–8 (8) µm long, present around lateral margins forwards from abdominal VI to head; extending across head, thorax and abdominal I and II, forming double to triple rows in each segment. Oral collar tubular ducts absent.

**Venter.** Ventral surface with normal flagellate setae present, mostly each 22–51 (30) µm long, many of them longer than those on dorsum, associated by shorter setae of different sizes, about 5–11 (11) µm long, except for longer setae on head, each about 65–152 (67) µm long. Trilocular pores present, each ca. 4 µm in diameter, similar to those on dorsum, scattered over venter. Multilocular disc pores small, each about 5–7 (6) µm in diameter, present around vulva only, few in number, totaling no more than 4. Oral rim tubular ducts, each with rim about 6–9 (9) µm in diameter and tube about 6–8 (6) µm long, similar to those on dorsum, each with rim wider than multilocular disc pores, distributed sparsely in marginal and submarginal zones, occasionally found in median zone. Oral rim tubular ducts absent from abdominal segment VIII. Oral collar tubular ducts about 5–7 (6) µm long and 2–3 (3) µm wide, present from abdomen III to VII, each segment with 4 to 8, located in submedial zone.

**Comments.** This new species comes close to *Chorizococcus kandyensis* (Green, 1922) from Sri Lanka (redescribed by Williams 2004). Both species possess dorsal oral rim tubular ducts extending across head, thorax and abdominal I and II, and ventral multilocular disc pores around vulva only. However, the new species possesses 3 pairs of cerarii (*Chorizococcus kandyensis* possesses only one pair) and oral rim tubular ducts of a single size on venter (*Chorizococcus kandyensis* possesses oral rim tubular ducts of two sizes), and lack of circulus (present in *Chorizococcus kandyensis*).

The adult female can be distinguished from all other instars by presence of a vulva opening between abdominal segments VII and VIII and by the presence of multilocular disc pores posterior to the vulva and oral collar tubular ducts on ventral abdominal segments.

It is unclear whether this new species is native or introduced from other country. According to approved facilities list for import of Bureau of Animal and Plant Health Inspection and Quarantine, Taiwan, no imports of Korean velvet grass(*Zoysia tenuifolia*) from other countries were recorded, therefore probably this species is native species to Taiwan. But this new mealybug species apparently occurs in a limited area in Taiwan, and only few parasitoid wasps were collected or observed around them, therefore it seems that this species was possibly introduced with its host plants, with other *Zoysia* species, or with other plant species.

##### Distribution.

Taiwan (Tainan City).

##### Etymology.

The specific epithet is based on the Latin genitive of the host-plant generic name *Zoysia*.

##### Description of the Third-Instar Female.

**Figure 3.**
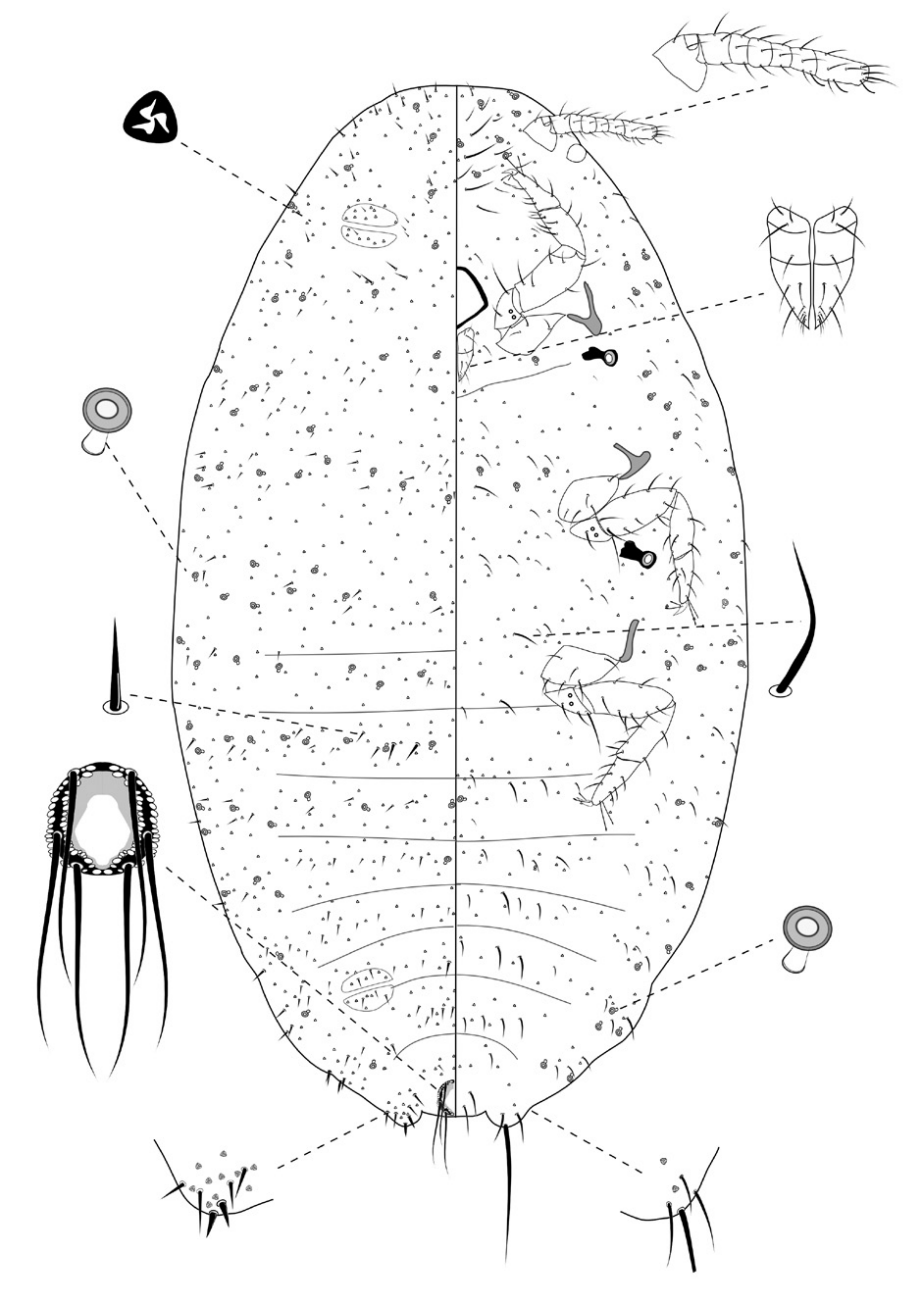
Third-instar female of *Chorizococcus zoysiae* Tsai, sp. n.

 **Slide-Mounted Features** **(measurements based on 13 specimens).** Body elongate oval, 1.06–1.50 mm long and 0.53–0.78 mm wide (Fig. 3). Antennae each 161–211 µm long, with 7 segments. Clypeolabral shield about 106–122 μm long. Labium about 83–106 µm long, shorter than clypeolabral shield. Legs well developed, stout; fore legs: coxa ca. 50–72 µm long, trochanter + femur 133–156 µm long, tibia + tarsus 128–150 µm long, ratio of lengths of tibia + tarsus to trochanter + femur 0.85–1.00, ratio of lengths of tibia to tarsus 1.01–1.18; mid legs: coxa ca. 56–94 µm long, trochanter + femur 150–164 µm long, tibia + tarsus 139–176 µm long, ratio of lengths of tibia + tarsus to trochanter + femur 0.89–1.02, ratio of lengths of tibia to tarsus 1.09–1.31; hind legs: coxa ca. 61–86 µm long, trochanter + femur 150–175 µm long, tibia + tarsus 150–172 µm long, ratio of lengths of tibia + tarsus to trochanter + femur 0.93–1.15, ratio of lengths of tibia to tarsus 1.11–1.50; claw stout, without denticle, about 20–21 µm long. Translucent pores absent. Both anterior and posterior ostioles present, not well developed, each lip with a few trilocular pores and 2–3 setae. Cerarii numbering usually 2 pairs, situated on posterior abdominal segments, occasionally 3 pairs. Anal lobe cerarii (C18) each bearing 2 slender conical setae, each seta about 16–22 µm long, with 2–6 stiff setae and 6–11 trilocular pores. Penultimate cerarii (C17), each containing 2 slender conical setae, each seta about 14–19 µm long, and 3–5 triocular pores. Antepenultimate cerarii (C16), if present, each bearing 2 slender conical setae, each seta about 11–18 µm long, and 2–5 trilocular pores. All cerarii on a membranous areas. Anal lobes moderately developed, each ventral surface membranous, bearing an apical seta 88–110 μm long. Anal ring about 33–45 µm long and 46–52 µm wide, bearing 6 setae, each seta about 79–92 µm long.

**Dorsum.** Dorsal surface with short, stiff setae, mostly each 10–28 µm long, accompanied by shorter setae of different sizes, about 4–7 µm long. Triocular pores present, each ca. 3 µm in diameter, scattered over dorsum. Multilocular disc pores absent. Oral rim tubular ducts, each with rim about 5–8 µm in diameter and tube about 5–8 µm long, present around lateral margins forwards from abdominal VII to head; extending across head, thorax and abdominal I to III, forming single row in each segment; usually only 1 on abdominal segments IV to VII.

**Venter.** Ventral surface with normal flagellate setae present, mostly each 13–43 µm long, many of them longer than those on dorsum, accompanied by shorter setae of different sizes, about 5–7 µm long, except for longer setae on head, each about 48–70 µm long. Trilocular pores present, each ca. 3 µm in diameter, similar to those on dorsum, scattered over venter. Multilocular disc pores absent. Oral rim tubular ducts, each with rim about 5–7 µm in diameter and tube about 6–8 µm long, similar to those on dorsum, distributed in marginal and submarginal zones, occasionally found in median zone, usually in groups of 4 to 7 on head and thorax and 2 or 3 on each side of each abdominal segment. Oral rim tubular ducts absent from abdominal segment VIII.

**Comments.** The third-instar female can be distinguished from the adult female by lacking a vulva, multilocular disc pores, and oral collar tubular ducts; and from earlier instars by bearing seven-segmented antennae.

##### Description of the Second-Instar Female.

**Figure 4.**
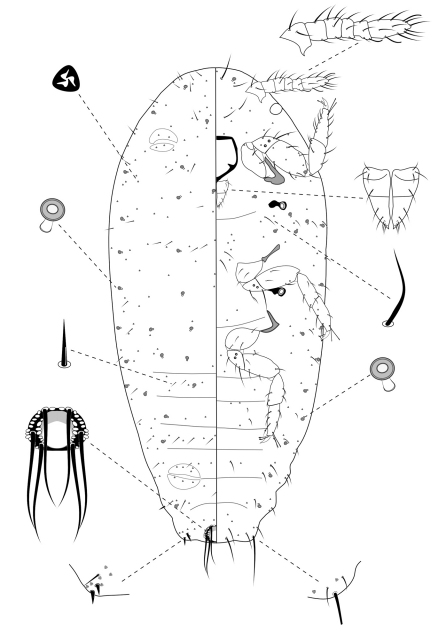
Second-instar female of *Chorizococcus zoysiae* Tsai, sp. n.

 **Slide-Mounted Features** **(measurements based on eight specimens).** Body elongate oval, 0.61–0.94 mm long and 0.38–0.44 mm wide (Fig. 4). Antennae each 133–161 µm long, with 6 segments. Clypeolabral shield about 78–94 μm long. Labium about 61–72 µm long, shorter than clypeolabral shield. Legs well developed, stout; fore legs: coxa ca. 39–44 µm long, trochanter + femur 103–111 µm long, tibia + tarsus 94–117 µm long, ratio of lengths of tibia + tarsus to trochanter + femur 0.89–1.08, ratio of lengths of tibia to tarsus 0.89–1.25; mid legs: coxa ca. 44–50 µm long, trochanter + femur 100–117 µm long, tibia + tarsus 111–117 µm long, ratio of lengths of tibia + tarsus to trochanter + femur 1.00–1.17, ratio of lengths of tibia to tarsus 1.00–1.41; hind legs: coxa ca. 44–50 µm long, trochanter + femur 111–117 µm long, tibia + tarsus 117–128 µm long, ratio of lengths of tibia + tarsus to trochanter + femur 1.05–1.15, ratio of lengths of tibia to tarsus 1.04–1.30; claw stout, without denticle, about 17–19 µm long. Translucent pores absent. Circulus absent. Both anterior and posterior ostioles present, not well developed, each lip with a few trilocular pores. Cerarii numbering usually 1 pairs on anal lobes. Anal lobe cerarii (C18) each bearing 2 slender conical setae, each seta about 14–16 µm long, with a stiff seta and 3–5 trilocular pores. All cerarii on a membranous areas. Anal lobes slightly developed, each ventral surface membranous, bearing an apical seta 75–94 μm long. Anal ring about 22–33 µm long and 39–44 µm wide, bearing 6 setae, each seta about 61–78 µm long.

**Dorsum.** Dorsal surface with short, stiff setae present, mostly each 5–13 µm long. Triocular pores present, each ca. 4 µm in diameter, scattered over dorsum. Multilocular disc pores absent. Oral rim tubular ducts, each with rim about 5–9 µm in diameter and tube about 6–7 µm long, present around lateral margins forwards from abdominal VII to head; extending across head, thorax and abdominal I to III, forming 4 longitudinal lines on marginal and submedial areas of head and thorax; usually only 1 on abdominal segments IV to VII. Oral collar tubular ducts absent.

**Venter.** Ventral surface with normal flagellate setae present, mostly each 9–33 µm long, many of them longer than those on dorsum, except for longer setae on head, each about 38–52 µm long. Trilocular pores present similar to those on dorsum, scattered over venter. Multilocular disc pores absent. Oral rim tubular ducts, each with rim about 6–8 µm in diameter and tube about 6–7 µm long, similar to those on dorsum, distributed in marginal zones, usually in groups of less than 6 on head and thorax and only one on each side of each abdominal segment. Oral rim tubular ducts absent from abdominal segment VIII. Oral collar tubular ducts absent.

**Comments.** The second-instar female can be distinguished from the third-instar female and adult female by its six-segmented antennae; and from the first-instar nymph by having oral rim tubular ducts.

##### Description of the First-Instar Nymph.

**Figure 5.**
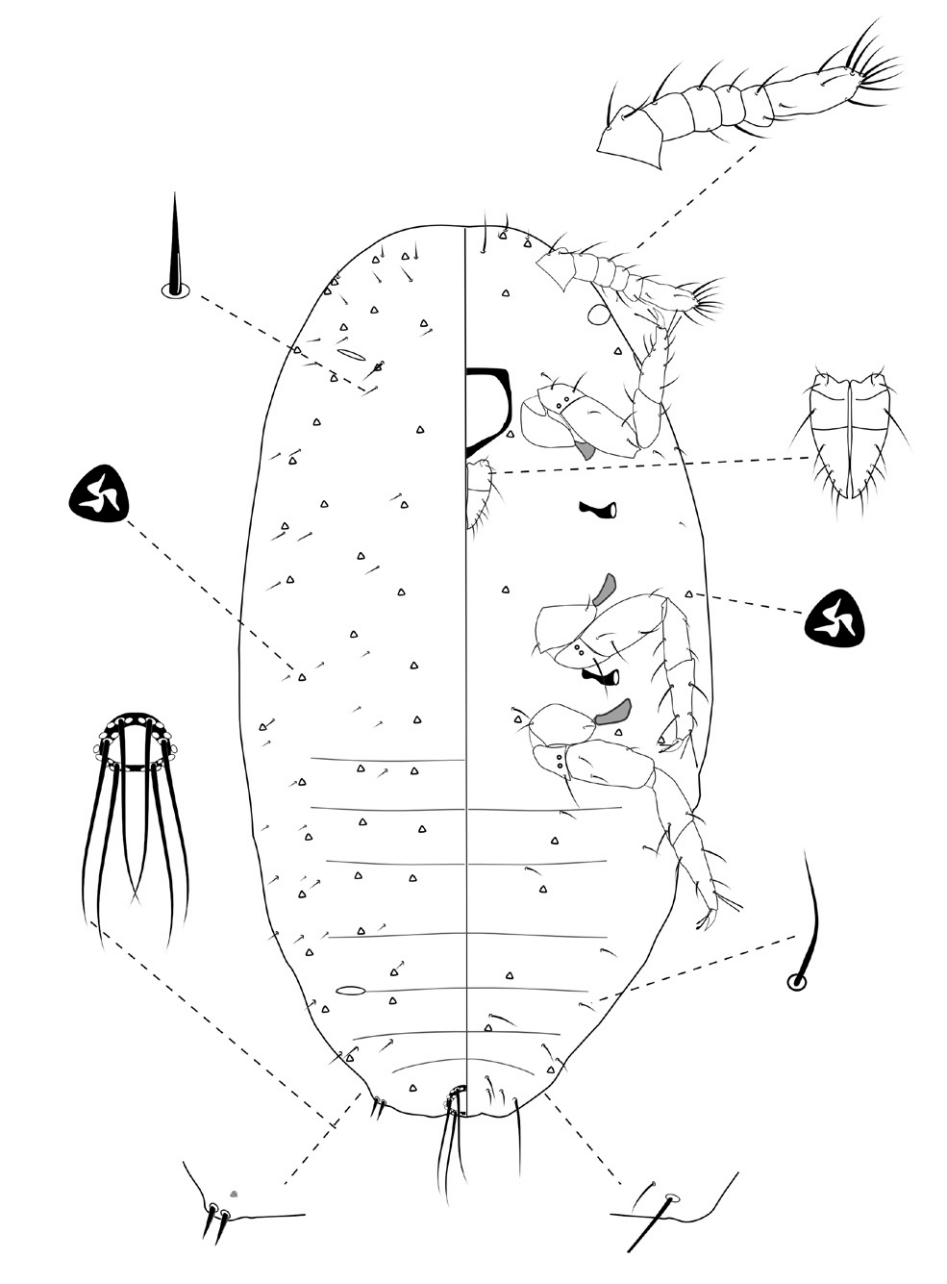
First instar nymph of *Chorizococcus zoysiae* Tsai, sp. n.

 **Slide-Mounted Features** **(measurements based on four specimens).** Body oval, 0.49–0.64 mm long and 0.21–0.32 mm wide (Fig. 5). Antennae each 100–128 µm long, with 6 segments. Clypeolabral shield about 61–72 μm long. Labium about 42–56 µm long, shorter than clypeolabral shield. Legs well developed, stout; fore legs: coxa ca. 28–33 µm long, trochanter + femur 67–83 µm long, tibia + tarsus 86–94 µm long, ratio of lengths of tibia + tarsus to trochanter + femur 1.07–1.42, ratio of lengths of tibia to tarsus 0.72–0.88; mid legs: coxa ca. 31–33 µm long, trochanter + femur 72–83 µm long, tibia + tarsus 92–100 µm long, ratio of lengths of tibia + tarsus to trochanter + femur 1.10–1.55, ratio of lengths of tibia to tarsus 0.78–0.89; hind legs: coxa ca. 31–33 µm long, trochanter + femur 72–89 µm long, tibia + tarsus 100–111 µm long, ratio of lengths of tibia + tarsus to trochanter + femur 1.13–1.54, ratio of lengths of tibia to tarsus 0.89–1.38; claw stout, without denticle, about 11–14 µm long. Translucent pores absent. Circulus absent. Both anterior and posterior ostioles present. Cerarii numbering one pairs, situated on anal lobes. Anal lobe cerarii (C18) each bearing 2 slender conical setae, each seta about 10–17 µm long, with 1 or 2 trilocular pores. All cerarii on a membranous areas. Anal lobes slightly developed, each ventral surface membranous, bearing an apical seta 56–75 μm long. Anal ring about 17–22 µm long and 28–33 µm wide, bearing 6 setae, each seta about 39–56 µm long.

**Dorsum.** Dorsal surface with short, stiff setae present, mostly each 6–10 µm long. Triocular pores present, each ca. 3 µm in diameter, scattered over dorsum. Multilocular disc pores absent. Oral rim tubular ducts absent. Oral collar tubular ducts absent.

**Venter.** Ventral surface with normal flagellate setae present, mostly each 11–19 µm long, many of them longer than those on dorsum, except for longer setae on head, each about 23–30 µm long. Trilocular pores present, each ca. 3 µm in diameter, similar to those on dorsum, scattered over venter in a few number. Multilocular disc pores absent. Oral rim tubular ducts absent. Oral collar tubular ducts absent.

**Comments.** The first-instar nymph can be distinguished from all instars by lacking oral rim and tubular ducts. It shares six-segmented antennae with the second instar, but its antennae are shorter than 130 µm (133–161 µm in second-instar females).

## Supplementary Material

XML Treatment for Chorizococcus

XML Treatment for Spilococcus mirzayansi

XML Treatment for Chorizococcus zoysiae
